# Health Benefits from Nature Experiences Depend on Dose

**DOI:** 10.1038/srep28551

**Published:** 2016-06-23

**Authors:** Danielle F. Shanahan, Robert Bush, Kevin J. Gaston, Brenda B. Lin, Julie Dean, Elizabeth Barber, Richard A. Fuller

**Affiliations:** 1 School of Biological Sciences, University of Queensland, Brisbane, Queensland, 4072 Australia; 2 School of Public Health, University of Queensland, Brisbane, Queensland, 4006 Australia; 3 Environment & Sustainability Institute, University of Exeter, Penryn, Cornwall TR10 9EZ, U.K; 4 CSIRO Land & Water Flagship, PMB 1, 107-121 Station Street, Aspendale, Victoria, 3195 Australia

## Abstract

Nature within cities will have a central role in helping address key global public health challenges associated with urbanization. However, there is almost no guidance on how much or how frequently people need to engage with nature, and what types or characteristics of nature need to be incorporated in cities for the best health outcomes. Here we use a nature dose framework to examine the associations between the duration, frequency and intensity of exposure to nature and health in an urban population. We show that people who made long visits to green spaces had lower rates of depression and high blood pressure, and those who visited more frequently had greater social cohesion. Higher levels of physical activity were linked to both duration and frequency of green space visits. A dose-response analysis for depression and high blood pressure suggest that visits to outdoor green spaces of 30 minutes or more during the course of a week could reduce the population prevalence of these illnesses by up to 7% and 9% respectively. Given that the societal costs of depression alone in Australia are estimated at AUD$12.6 billion per annum, savings to public health budgets across all health outcomes could be immense.

Urbanization is emerging as one of the most important global health issues of the 21^st^ century^1^,^2^, with cities becoming epicenters for chronic, non-communicable physical and mental health conditions^3^,^4^. There is growing recognition of the crucial role of urban green spaces in addressing this public health challenge^5^,^6^, with over 40 years of research showing that experiences of nature are linked to a remarkable breadth of positive health outcomes. This includes improved physical health (e.g. reduced blood pressure^7^ and allergies^8^, lower mortality from cardio-vascular disease^9^, improved self-perceived general health^10^,^11^), improved mental wellbeing (e.g. reduced stress^12^ and improved restoration^13^,^14^), greater social wellbeing^15^, and promotion of positive health behaviors (e.g. physical activity^16^,^17^). Consequently, cities across the world are investing in the provision, management and enhancement of public green spaces, with the 100 largest cities in the US alone spending over US$6 billion in 2015^18^. Advice about how to achieve health outcomes from green spaces currently remains very general^19^,^20^. Evidence on how frequent or how long nature experiences need to be, or what types of nature are needed, is vital to ensure that investment in green space provision can cost-effectively help to meet the public health challenges of urbanization^21^,^22^,^23^.

Here, for the first time we use the nature-dose framework posed by Shanahan *et al*.^21^ to quantify the link between health outcomes and experiences of nature, as measured by *intensity* (i.e. the quality or quantity of nature itself), and the *frequency* and *duration* of a city resident’s experiences. We focus on examples of health issues across four domains for which there is some prior evidence that nature exposure can provide benefits. These health issues are also particularly relevant for cities, and include mental health (the prevalence of depression), physical health (high blood pressure), social wellbeing (social cohesion), and a positive health behaviour (physical activity). These health outcomes could be tied to experiences of nature through a range of mechanistic pathways (some of which are outlined in Fig. 1)^22^. For example, a higher level of vegetation within a landscape (a measure of nature intensity) may be linked to enhanced physical, mental and social wellbeing through providing a visually complex environment that can lead to reduction in stress^24^, reduction of mental fatigue^25^, or by adding to the look and feel of a place and so providing a pleasant location for social or physical activities^22^ (Fig. 1). Similarly, variation in duration and frequency of nature exposure could also influence the long-term health outcomes people experience, with even short-duration exposure to natural environments shown to deliver an immediate reduction in blood pressure^7^ and greater feelings of restoration^26^. Yet despite this, whether and how the intensity, frequency or duration of nature exposure leads to long-term and lasting effects on health remains unexplored.

Unpacking the relationship between health outcomes and the three components of nature dose also allows for the exploration of dose-response relationships, including whether there is a minimum dose where some effect of natire on health might be seen^21^,^27^. Here we therefore use dose-response modelling to determine how rates of high blood pressure and depression vary in response to nature experiences, including whether the outcomes plateau or continue to improve^21^. We examine the scale of the population health benefits that could arise if these nature dose recommendations are met, and the impact of this on the public health purse.

## Results

The first stage of our analysis was to examine the relationship between individual-level experiences of nature and four health outcomes in a population sample of 1538 residents of Brisbane City, Australia. These health outcomes included whether the respondent scored as having mild or worse depression determined from an established 7 item questionnaire^28^, whether the respondent reported being under treatment for high blood pressure, perceptions of social cohesion derived from three survey questions^29^,^30^,^31^, and the self-reported number of days on which physical exercise occurred for more than 30 minutes during the survey week.

We measured experiences of nature across three components, including the usual frequency of outdoor green space visits across a year, the average duration of visits to green space across a week, and the intensity of nature (measured as the highest level of vegetation complexity within any of the green spaces that a respondent visited, following a hypothesis that higher levels of vegetation lead to greater health outcomes; Table 1, Fig. 2). Multivariate analyses revealed that a longer duration of individual nature experiences was significantly linked to a lower prevalence of depression and of high blood pressure, and increased physical activity. A higher frequency of green space visitation was an important predictor for increased social cohesion, and both duration and frequency showed a significant positive relationship with higher levels of physical activity (Table 1). These multivariate analyses accounted for key covariates including age, gender, Body Mass Index (BMI; weight in kilograms/square of height in meters), and socio-economic indicators including the income, education, and neighborhood socio-economic disadvantage (Index of Socio-economic Disadvantage, IRSD; Table 1)^32^. We also found that people with a stronger self-reported connection to nature (measured using the Nature Relatedness scale^33^) had greater levels of social cohesion and physical activity, but did not show a reduced prevalence of depression or high blood pressure (Table 1).

We examined the dose-response relationship between the odds of a respondent being recorded as having high blood pressure or depression and incremental increases in the duration of nature experiences, while accounting for covariates (Fig. 3, Table 2). We found that the odds were significantly lower than the null model for depression when reported green space visits were an average of 30 minutes or more (i.e. the confidence interval did not overlap with an odds ratio of one; Fig. 3A), with a slight increase in mean gains until a duration of 1 hour 15 minutes. For high blood pressure, there was also a significant health improvement after 30 minutes of exposure, though the dose-response curve showed high variability at higher exposure levels (Fig. 3B). The power of the test for high blood pressure and depression was reduced at higher durations (indicated by wider 95% confidence intervals).

We found that the proportion of cases of depression and high blood pressure in the population that can be attributed to city residents failing to spend an average of 30 minutes or more during a green space visit across the course of their week (the ‘population attributable fraction’) was 0.07 for depression, and 0.09 for high blood pressure (Table 2); that is, there could be up to 7% fewer cases of depression and 9% fewer cases of high blood pressure if the entire sampled population met the minimum duration criteria of 30 minutes or more.

## Discussion

The results here suggest that nature experiences in urban green spaces may be having a considerable impact on population health, and that these benefits could be higher if more people were engaged in nature experiences. Specifically, our results suggest that up to a further 7% of depression cases and 9% of high blood pressure cases could be prevented if all city residents were to visit green spaces at least once a week for an average duration of 30 minutes or more. The societal costs of depression are estimated at AUD$12.6 billion per annum for employed Australians alone^34^, and the direct costs of hypertension in the United States have been estimated at US$48 billion^35^. Given that our results show nature experiences, if causal in nature, could simultaneously lead to a suite of health benefits for mental health (depression), physical health (high blood pressure), social health (social cohesion), and a positive health behavior (physical activity), the cumulative cost savings across all health outcomes could be immense if this behavioral change was targeted.

Our finding that the duration, and frequency of nature interactions are varyingly associated with the four health outcomes has potentially important implications for the design of health interventions, and also reveals new hypotheses that warrant further attention. For example, while provision and quality of green spaces is undoubtedly important, health programs aiming to reduce the prevalence of depression or high blood pressure might also focus on behavioral interventions, for example, promoting longer duration green space visits. In contrast, improved social cohesion in communities is a well-known benefit of public green spaces^36^,^37^, and interventions that aim to enhance social cohesion might fruitfully focus on increasing residents’ frequency of visits^38^. Social cohesion is itself important for public health, as it is positively associated with physical and mental wellbeing^39^. These flow-on benefits are likely to add considerably to the economic and social value of urban green space.

Here physical activity was associated with both higher duration and frequency of green space visits, which is important given it can reduce the risk of a wide range of non-communicable diseases such as diabetes, cardiovascular disease and obesity^40^. Green spaces are often considered settings that directly facilitate exercise^41^, and visiting green spaces can incidentally entail walking, running or cycling. Vegetated areas also offer shade and improved temperature regulation^42^, providing a pleasant location for physical activity. This is particularly relevant in cities such as Brisbane, a sub-tropical location with hot summers and a mean of 113 cloudless days per year^43^. However, while many studies have found that more people undertake physical activity (e.g. cycling and walking) in greener neighbourhoods^17^, the results are sometimes mixed; for example, these patterns could be due to other activities such as gardening^44^, or because active people self-select into greener neighbourhoods^45^. While our results add to the body of knowledge on this subject, these varying explanations require further attention.

Our measure of nature intensity (vegetation complexity) showed no association with any of the health outcomes measured. Other studies have found that higher levels of plant, butterfly and bird species richness (or perceived species richness) can enhance a person’s feelings of restoration^13^,^14^, and future work might fruitfully explore the effect of such measures within the nature dose framework. There are also other hypotheses describing relationships between health and vegetation complexity; for example, studies have found that more people tend to visit public green spaces with moderate levels of vegetation cover (rather than high or low)^46^, and vegetation is also likely to influence the perception of safety of an area^25^. Systematic consideration of nature dose-response relationships will therefore be critical to understanding how to enhance health outcomes from exposure to nature.

We observed significantly fewer cases of depression and high blood pressure in people who spent an average of 30 minutes or more visiting green space in the survey week, and there was some indication that longer duration visits may be associated with an even lower prevalence of depression. However, here we traded-off accuracy in detecting differences across the incremental increases in dose for achieving a high level of representation across the population (i.e. sampling did not target respondents with varying durations of nature exposure). Given that this type of dose-response relationship could contribute further evidence for causality according to Hill’s criterion^47^, future studies would benefit from achieving relatively even sampling representation across the relevant nature dose levels. An added consideration when interpreting the results outlined here is that the effects of depression itself can influence a person’s activity levels^48^, and so could reduce the likelihood that a person visits green-space. The same effect could also occur for high blood pressure, where people who have other risk factors such as obesity might also be less likely to visit green spaces (note, BMI and physical activity were considered as covariates here, so these effects are somewhat accounted for). Thus, studies that explore changes over time within individuals and across populations could be a particularly powerful way to further elucidate dose-response relationships between nature and health.

This study used a self-report online survey, an approach which brings a number of benefits (such as the large sample size and a high level of stratification across the population), as well as limitations. For example, recalling events can pose challenges, question order can affect responses, and many other factors can affect how well a person responds to questions^49^. While we used measures to minimize these limitations, other methods such as longitudinal studies using tracking technologies might provide complementary understanding of nature-dose relationships. Future research exploring the role of a broader range of socio-demographic and community factors related to health outcomes, but which also have the potential to influence interaction with nature (e.g. marital status and crime) will also shed light on the mechanistic pathways linking nature exposure to health.

Nature relatedness, or the differences in the way people view their connection with the natural world, could both drive interactions with nature and enhance wellbeing in its own right^50^. We found that higher levels of nature relatedness predicted greater feelings of social cohesion and higher levels of physical activity. This supports other research which has found that people with higher nature relatedness scores also often report better wellbeing, happiness and life satisfaction^33^,^51^, and lower levels of anxiety^52^. A limitation of studies so far within this area is that they are often single time-point studies, and research is needed to whether actively altering this trait might influence health and wellbeing.

Interactions with nature simultaneously deliver mental, physical and social health outcomes for a population through multiple pathways^22^. By harnessing the synergistic potential of these pathways, contact with nature has the potential to lower not just the prevalence of single chronic conditions, but also multiple chronic or acute medical conditions that co-occur within one person. However, here we have also shown that the different components of experiences of nature (the frequency, duration or intensity) variously influence the health outcomes. This has important implications for the design of health interventions targeting improvements in the four health domains examined here. Ongoing efforts to unpack the nature-health relationship will be vital to combat the emerging public health challenges associated with urbanization, and to ensure that investment in green space provides value for money^21^,^22^,^23^.

## Materials and Methods

### Survey

This research was conducted in accordance with approved guidelines, and all protocols were received Institutional Human Research Ethics Approval (Behavioural & Social Sciences Ethical Review Committee, University of Queensland), project number 2012000869. Informed consent was obtained from all respondents. The full survey is available in the Supplementary material.

We surveyed 1538 Brisbane residents aged 18–70 years to obtain information on health and experiences of nature. The survey was delivered online by Q&A Market Research Ltd to their existing market research database of potential respondents, and carried out in November 2012. This time period was chosen as it is prior to the onset of higher summer temperatures, ensuring that the outcomes were minimally affected by seasonal conditions and because it is prior to the summer holiday period which could also affect participation and the measured behaviors^53^. Brisbane City has high overall levels of public green space (>200 m^2^ per person) and tree cover (36%), both of which are spread rather evenly across the socio-economic gradient^54^. Thus baseline exposure to nature outside of the experiences measured in this study (i.e. through day-to-day activities at home or work) is likely to be high across city residents.

The respondent group was recruited based on whether they fulfilled a number of stratification criteria across a range of factors, which ultimately ensured that the socio-demographic distribution closely reflected that of the actual population (Table S1), according to age (similar numbers above and below 45), sex (similar numbers of males and females), income quartiles within the city, and respondents’ addresses were spread evenly among four spatial zones reflecting the four quartiles of tree cover across the city (Figure S1). A Pearson’s rank sum test was conducted to compare the proportion of representation within the different stratification criteria against that of the real population, and showed that the characteristics of the surveyed population were well correlated with that of the actual population (correlation coefficient = 0.67, t = 7.14, p < 0.0001).

Socio-demographic variables that are tied to health outcomes were collected, including age, sex, personal annual income, highest formal qualification, presence of children under 16 in the home, the primary language spoken at home, and number of days the respondent normally spends at work per week. Respondents also provided information on their height and weight, from which we calculated BMI. The Australian census-derived Index of Relative Socio-economic Disadvantage (IRSD) was used as a measure of the level of socio-economic disadvantage in the respondent’s neighborhood, calculated for the finest possible spatial scale (Statistical Area 1, mean area = 0.44 km^2^,^55^). We also measured a person’s connection to nature using the Nature Relatedness scale^33^, as this could moderate any benefits gained from experiences of nature. All variables are described in detail in Table 3.

### Experiences of nature

Respondents were invited to report on any visit within the previous week to a place they considered ‘outdoor green space’, and were asked to name or describe the location. We manually geo-located these locations based on the descriptions where possible. Three aspects of nature dose were measured, encompassing the duration and frequency of experiences, and nature intensity, through a mixture of self-report and remote sensing analysis. Nature dose questions were asked in the survey before the health questions to avoid any potential priming effects of a person’s health status on self-reported nature dose (e.g. see^49^).

#### Duration of experiences of nature

Average duration of green space visits was estimated based on self-reported time spent during each visit across the survey week. We chose this timeframe as it provided a short and recent reference period to improve accuracy^49^. Note that this measure of duration is necessarily linked to frequency, as to achieve a duration measure the respondent must have visited a green space at least once during the survey week. Duration was selected from a time category (1–29 minutes; 30 minutes to one hour; one to two hours; two to three hours; three to four hours; four or more hours), and the mid-point of each selected category was summed (with four or more hours being treated as ‘four’), and this value was averaged across all visits.

#### Frequency of experiences of nature

Given that frequency of visitation would be highly correlated with duration if measured on the same time scale, here it was estimated based on the respondent’s self-reported frequency of visits to green spaces where their usual frequency of visits across a year was selected from the following categories: never; once a year; once every three months; two to three times a month; once a month; once or more per week. This approach also allowed us to account for people who use green spaces infrequently (i.e. less than once a week who were missed by the duration measure).

#### Nature intensity

**Figure 1:**
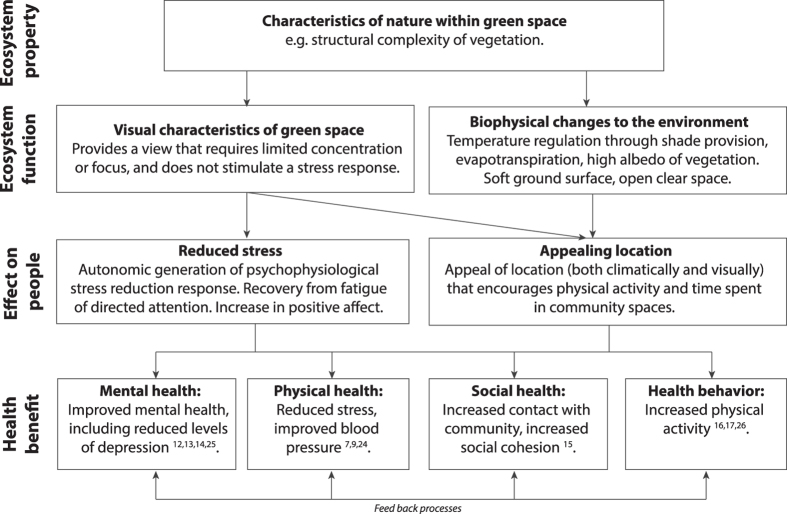


**Figure 2:**
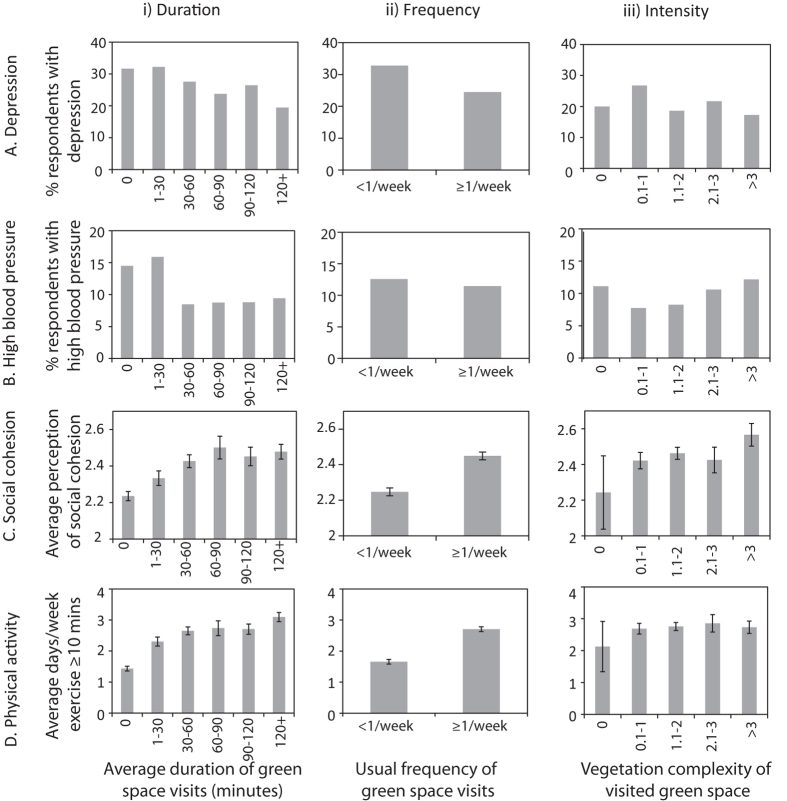
The bivariate relationships between health responses (**A–D**) and nature experiences, comprising (i) the average duration of visits to green space; (ii) the normal reported frequency of visits to green space; and (iii) the nature intensity, measured as vegetation complexity within the best visited public green space. Error bars are standard errors.

**Figure 3:**
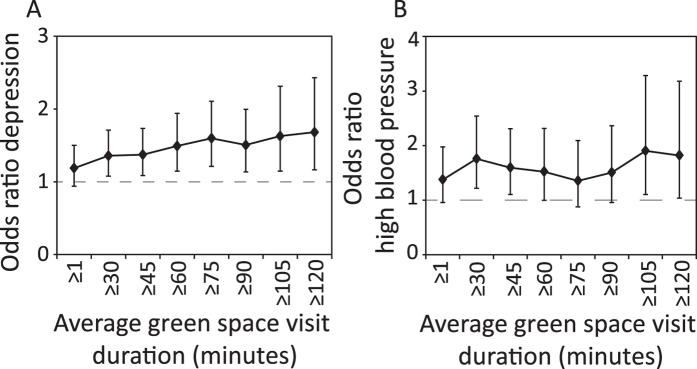
Dose-response graphs showing the adjusted odds ratio from logistic regression for incrementally increasing average duration of green space visits. 95% confidence intervals are shown. An odds ratio above one indicates an individual is more likely to have the disease where the threshold of green space visitation is not met.

Here we generated one possible measure of nature intensity, the vegetation complexity within the most complex map-able green space each respondent visited (hypothesizing that more complex vegetation leads to better health outcomes by promoting attention restoration, and increasing the appeal of green spaces; Fig. 1; this measure also tends to correlate with plant and animal diversity^56^,^57^). Most (77%) of respondents only visited one or two green space locations so other measures such as the most common, or average complexity were not useful here. Analyses involving nature intensity were limited to respondents for whom the visited green space (a) could be geo-located, and (b) had established boundaries within the Brisbane City limits to ensure we vegetation was measured within the visited area. Complexity was measured using LiDAR-derived maps of vegetation cover at a 5 × 5 m resolution (details provided in the Supplementary material). Five separate vegetation strata were used that have relevance to the human experience of nature, including 0.15–1 m (likely to influence access and egress); 1–2 m (the line of sight may be affected); and three layers likely to provide varying levels of shade and visual vegetation complexity, 2–5 m; 5–10 m; 10 m+. For each of the vegetation strata we created a binary grid layer (where 1 indicated vegetation was present), and we summed all five of these layers for each 5 × 5 m pixel. We calculated the average summed measure across the entire green space. Higher values of vegetation complexity were achieved in green spaces with higher vegetation cover and more complex vegetation structure. This measure was calculated for 664 survey respondents who visited green spaces within the study area, and only these respondents were used in relevant analyses.

### Health response measures

Respondents provided information on four health outcomes:

#### Mental health

A measure of depression was generated based on the depression component of the Depression, Anxiety and Stress scale^28^. Scores were converted to a binary measure where 0 indicates no depression and 1 indicates mild or worse depression.

#### Physical health

Respondents reported whether they were currently receiving treatment for high blood pressure, coded as a binary measure where 0 indicates no treatment and 1 indicates treatment.

#### Social health

Respondent’s perceptions of social cohesion were estimated based on three previously developed questions that measure trust, reciprocal exchange within communities, and general community cohesion^29^,^30^,^31^ (see Supplementary material for details). The scores across all three questions were averaged.

#### Health behavior

Respondents provided a self-report indication of physical activity, specifically the number of days they exercised for 30 minutes or more during the survey week (regardless of location; ‘green exercise’ and exercise in other locations were not differentiated). The resulting count variable was between 0 and 7.

### Statistical Analyses

All analyses outlined here were conducted in the software package R^58^. We used an exploratory approach to examine the correlation between each health response and potential predictors (outlined in detail in Table S1), including socio-demographic variables, BMI, physical activity (where it was not also the response variable), and the three nature experience measures. We used generalized linear models (binomial) for depression and high blood pressure, linear regression models for social cohesion, and negative binomial generalized linear models for physical activity. The three measures of nature dose were correlated (significant Spearman’s rank test correlations of 0.50–0.57), so to avoid issues associated with multicollinearity we generated four predictor model sets for each health response: (i) all socio-demographic variables (but excluding the frequency, duration and intensity of nature experiences); (ii) socio-demographic variables plus duration of nature experiences; (iii) socio-demographic variables plus frequency of nature experiences; (iv) socio-demographic variables plus nature intensity. Neighborhood socioeconomic disadvantage (IRSD) was reverse square-root transformed and BMI was log transformed to ensure models met assumptions of normality. We calculated the model averaged coefficient estimates for each predictor variable by generating models with all possible variable combinations, and averaged the coefficient for each across all models in which it was present (using the R package MuMln).

To further explore any relationships which became evident from the analyses above, we conducted dose-response modelling for the two binary health measures (depression and high blood pressure) where there was evidence for an effect of any one of the three nature dose variables. Dose response modelling is readily achieved for binary response variables^40^; social cohesion and physical activity did not lend themselves readily to this analytical approach because there is no threshold where a score is ‘good’ or ‘bad’. To carry out this approach we first built a logistic regression model where the predictor variables were treated as ‘risk factors’, an established practice in population epidemiology^59^,^60^. The relative odds of occurrence of either depression or high blood pressure in an individual were calculated given a person’s specific risk factors (e.g. age) or duration, frequency or intensity of nature experiences. We used only the predictor variables that were statistically significant in the analysis in Table 1, and transformed each into a binary risk factor using existing evidence where possible. For example, for age the risk of being diagnosed with hypertension begins to increase steeply at age 45 years^61^, and the prevalence of affective mood disorders such as depression begins to decline in Australia at about 45^62^. We therefore used 45 years to create a binary risk factor above which the risk of having depression was zero, and below one (and vice versa for high blood pressure). Similarly, Australian guidelines recommend physical activity on most, if not all days per week^63^, and we therefore created a binary risk factor as people who exercised for 30 minutes on 5 days or more (0) and those who did not (1). Respondents who were ‘overweight’ (≥25 BMI^64^) were categorized as a risk factor of 1, and those under as 0. Where no definitive information was available we used the results from Table 1 to guide the direction of the risk categorization; this includes whether children were present in the home, whether a person works (treated as a binary work or no-work), and income and neighborhood disadvantage (IRSD; with the binary categorization reflecting whether the respondent fell into the top half or bottom half of the population values). Variables for which no threshold could be estimated were omitted from these analyses (as was the case for social cohesion and nature relatedness).

To create a dose-response curve, we ran the logistic regression models described above with incrementally increased thresholds of nature experiences (e.g. for duration a person’s risk factor was varied based on whether they met incremental thresholds including >0 minutes; ≥15 minutes; ≥30 minutes; ≥45 minutes; ≥1 hour and so forth until the maximum time of 4 hours), and determined the odds ratio that a person who fell within that category would have the condition. We identified the point at which health gains were first recorded as better than the null model on plots of nature dose versus the odds ratio for use in the analysis described below.

A population average attributable fraction analysis was used to estimate the proportion of depression and high blood pressure cases in the population attributable to each of the predictor variables or ‘risk factors’^60^. Within a multivariate logistic regression environment, each risk factor was removed sequentially from the population by classifying every individual as unexposed (i.e. risk factor of 0). The probability of each person having the disease was then calculated, where the sum of all probabilities across the population was the adjusted number of disease cases expected if the risk factor was not present. The attributable fraction was calculated by subtracting this adjusted number of cases from the observed number of cases. The risk factors were removed in every possible order, and an average attributable fraction from all analyses was obtained.

## Additional Information

**How to cite this article**: Shanahan, D. F. *et al*. Health Benefits from Nature Experiences Depend on Dose. *Sci. Rep.* **6**, 28551; doi: 10.1038/srep28551 (2016).

## Supplementary Material

Supplementary Information

Supplementary Information

## Figures and Tables

**Table 1:** The relationship between four health outcomes (the response variables), socio-demographic covariates and nature experience predictor variables.

Predictor variables	Depression	High blood pressure	Social cohesion	Physical activity
**Model (i)**	**Pseudo R**^**2**^** = 0.10**	**Pseudo R**^**2**^** = 0.41**	**R**^**2**^** = 0.10**	**Pseudo R**^**2**^** = 0.05**
Age	−0.02 (0.01)***	0.12(0.01)***	0.01(0.00)***	−0.01(2e-3)***
Gender	−0.31(0.12)*	−0.03(0.19)	−0.08(0.03)*	−0.08(0.06)
Income	−0.00 (0.00)*	0.00 (0.00)	0.00(0.00)	0.00(0.00)
Children in home	−0.10 (0.07)	0.32 (0.12)**	0.11(0.02)***	−0.10(0.03)**
Neighborhood disadvantage	−0.03(0.02)	−0.06 (0.03)*	0.03(0.005)***	0.03(9e-3)**
Work days/week	−0.07(0.03)*	−0.04 (0.04)	0.02(0.01)*	0.00(0.01)
Highest qualification	−0.00 (0.05)	0.04 (0.08)	−0.00(0.01)	0.04(0.03)*
Ethnicity	−0.16(0.18)	0.47(0.33)	0.013(0.04)	0.03(0.08)
Physical activity frequency	−0.13(0.03)***	0.06 (0.04)	0.03(0.01)***	NA
BMI	1.28(0.29)***	3.67 (0.46)***	−0.04(0.07)	−0.07(0.10)
Social cohesion	−0.42(0.10)***	−0.28(0.16)	0.17(0.03)***	0.15(0.05)**
Nature relatedness	−0.06 (0.10)	−0.07 (0.16)	0.01(0.00)***	0.20(0.05)***
**Model (ii)**	**Pseudo R**^**2**^** = 0.10 n = 1538**	**Pseudo R**^**2**^** = 0.42 n = 1538**	**R**^**2**^** = 0.11 n = 1538**	**Pseudo R**^**2**^** = 0.08 n = 1538**
+ Nature experience duration	−0.16 (0.06)*	−0.23(0.1)*	0.11(0.03)***	0.19(0.03)***
**Model (iii)**	**Pseudo R**^**2**^** = 0.10 n = 1538**	**Pseudo R**^**2**^** = 0.41 n = 1538**	**R**^**2**^** = 0.12 n = 1538**	**Pseudo R**^**2**^** = 0.0.06 n = 1538**
+ Nature experience frequency	−0.06(0.04)	0.09 (0.09)	0.16(0.02)***	0.16(0.01)***
**Model (iv)**	**Pseudo R**^**2**^** = 0.10 n = 664**	**Pseudo R**^**2**^** = 0.41 n = 664**	**R**^**2**^** = 0.10 n = 664**	**Pseudo R**^**2**^** = 0.0.08 n = 664**
+Nature experience intensity	−0.16(0.10)	0.29 (0.02)	0.00(0.02)	0.00(0.08)

Four models for each response variable are shown: (i) socio-demographic variables only; (ii) socio-demographic variables plus duration of nature experiences; (iii) socio-demographic variables plus frequency of nature experiences; (iv) socio-demographic variables plus nature intensity. Model averaged coefficients are shown with standard error in brackets, and the Nagelkerke/Crag and Uhler’s pseudo R^2^. Positive coefficients indicate rates of depression and high blood pressure were higher with higher values of the predictor variables, and that social cohesion and physical activity increased. Significance: *p < 0.05; **p < 0.01; ***p < 0.001.

**Table 2:** The odds ratios for a person having depression or high blood pressure where specific risk factors are present (the result for each variable was calculated while accounting for all their other risk factors; i.e. multivariate analyses), and the proportion of disease cases in the study population attributable to various risk factors (average population attributable fraction).

	Depression: Risk factor	Odds ratio (95% confidence intervals)	Average attributable fraction	High blood pressure: Risk factor	Odds ratio (95% confidence intervals)	Average attributable fraction
Age	Higher risk ≤45 years	1.62(1.25,2.09)	0.13	Higher risk ≥45 years	16.56(9.71,28)	0.44
Gender	Higher risk for males	1.31(1.05,1.65)	0.07	*NA*		
Children	*NA*			Higher risk with children	2.02(1.27,3.21)	0.04
Income	Higher risk for bottom half of population	1.33(1.05,1.7)	0.06	*NA*		
Neighborhood disadvantage	*NA*			Higher risk for bottom half of population	1.5(1.05,2.15)	0.06
Work	Higher risk for non-workers	1.47(1.12,1.95)	0.05	*NA*		
Physical activity	Higher risk for those that exercise for <5 days/week	2.05(1.46,2.89)	0.27	Higher risk for those that exercise <5 days/week	0.81(0.50,1.29)	
BMI	Higher risk BMI >25	1.28(1,1.62)	0.06	Higher risk BMI >25	4.34(2.76,6.81)	0.28
Nature experience duration	Higher risk where duration of visits <30 minutes	1.37(1.09,1.74)	0.07	Higher risk where duration of visits <30 minutes	1.76(1.21,2.53)	0.09

An odds ratio above 1 indicates the disease is more likely to be present where the risk factor is present. n = 1538.

**Table 3:** Descriptions of the variables tested for correlation with each of the four health responses.

Variable name	Description
Age	Respondent’s age in years, selected from 11 categories.
Gender	Gender, for analysis purposes male = 0, female = 1.
Income	Personal income selected from categories defined based on the income question provided in the Australian census (categories included weekly income of: nil or negative; $1-$199; $200-$299; $300-$399; $400-$599; $600-$799; $800-$999; $1000-$1249; $1250-$1499; $1500-$1999; $2000+). For analysis purposes the lowest value of the income bracket indicated by respondent was used, and variable was treated as numeric ordinal.
Neighborhood disadvantage	The Index of Socioeconomic Disadvantage (IRSD), a census derived indicator provided by the Australian Bureau of Statistics was used. Variable is continuous (between 650–1150 in this sample), with low scores indicating greater deprivation. The neighborhood value for each respondent’s address was used at the finest available spatial scale (Australian Census Statistical Area 1).
Children living at home	The presence or absence of people living in a respondent’s home who were under 16 years at the time of the survey.
Work days per week	Number of days the respondent works in an average week.
Highest qualification	The highest formal educational qualification achieved by the respondent, grouped into five categories (5 = highest qualification possible, e.g. post-graduate qualification; 1 = lowest qualification possible, e.g. year 10 of school).
Language (non-English = 1)	An indication of the language primarily spoken at home. For analysis purposes 0 = English, 1 = not English.
Frequency of physical activity	Number of days the respondent carried out physical activity for 30 minutes or more.
BMI	Respondent’s Body Mass Index (BMI), weight in kilograms divided by height in meters squared.
Social cohesion	Score to indicate perceptions of social cohesion derived from three questions, described in detail in the Supplementary Material.
Green space visitation frequency	Ordinal variable indicating the self-reported frequency of visits to public green spaces selected from categories, including: never; once a year; once every three months; once a month; 2–3 times a month; once or more per week. Ordered numeric variable.
Green space visitation duration	Average time spent during each visit to public green spaces reported for the survey week. Ordered numeric variable.
Green space visitation intensity	The ‘volume’ of vegetation within the most heavily vegetated green space visited by each respondent. The variable was calculated by estimating average vegetation volume from five structural layers across the entire green space. Green spaces with the most structurally complex vegetation across large areas score highest. Continuous variable.
