# Supplementary material for: Health Benefits from Nature Experiences Depend on Dose
Source: Sci Rep. 2016 Jun 23;6:28551. doi: 10.1038/srep28551 (PMC4917833; doi:10.1038/srep28551)
Supplement: Supplementary Information [file srep28551-s1.pdf]

SUPPLEMENTAL INFORMATION -GENERAL

Manuscript title: Health Benefits from Nature Experiences Depend on Dose

Authors: Shanahan, D.F., Bush, R., Gaston, K.J., Lin, B.B., Dean, J., Barber, E., Fuller, R.A.

Similarity between the demographic distribution of the survey data compared with the Australian 2011 census data

Table S1. Ratio of the percentage of survey respondents within each socio-demographic category to the percentage within that group within the actual population as shown by the Australian census [1]. The green space zones represent the four quartiles of tree cover present within postcode areas across the city, with zone 1 being the lowest cover and zone 4 the highest.

	Income quartile	Males ≥18, ≤45 years	≥46, ≤70 years	Females ≥18, ≤45 years	≥46, ≤70 years	Overall
**Green space zone 1**	**1**	1.22	1.87	0.9	2.07	**1.17**
	**2**	1.39	1.86	0.93	1.48	**1.32**
	**3**	3.12	1.8	1.29	1.12	**1.44**
	**4**	0.66	1.63	1.21	1.03	**1.00**
**Green space zone 2**	**1**	0.62	0.82	0.61	0.71	**0.66**
	**2**	1.1	1.93	0.52	1.31	**1.08**
	**3**	1.36	0.84	1.29	1.34	**1.23**
	**4**	1.09	0.61	2.3	0.63	**1.02**
**Green space zone 3**	**1**	0.91	1.24	0.85	1.23	**1.00**
	**2**	1.65	1.76	0.9	1.11	**1.25**
	**3**	1.6	0.79	0.75	0.38	**0.93**
	**4**	0.86	0.8	1.03	0.49	**0.80**
**Green space zone 4**	**1**	2.75	1.1	1.1	1.15	**1.32**
	**2**	1.54	2.16	1.43	1.17	**1.51**
	**3**	1.48	0.6	1.02	1.28	**1.14**
	**4**	0.82	1.31	1.87	1.73	**1.25**
**Overall**		**1.25**	**1.07**	**1.00**	**1.05**	

Figure S1. Map showing all the postcodes of Brisbane, Australia, categorized into four spatial zones based on percentage cover of tree cover (tree cover dataset described in main manuscript). Each postcode was categorized based on the quartile of percentage tree cover it fell within (zone one is the lowest tree cover quartile, zone four is the highest). The survey sampling strategy achieved even levels of representation within the four quartiles. Map was created in ArcGIS V10.3 (www.ArcGIS.com).

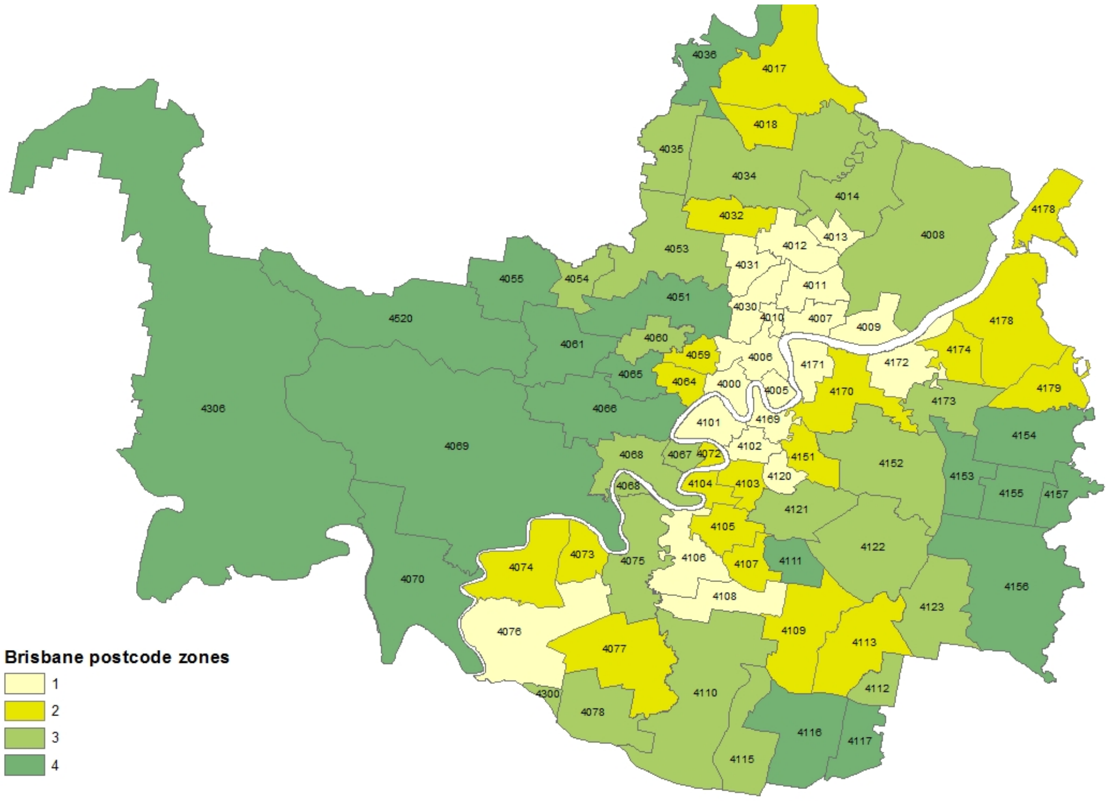

Development of social cohesion measure

We generated estimates of each respondent's perception of social cohesion using three questions that provided an indication of trust, reciprocal exchange within communities, and general community cohesion. The first question was a social cohesion and trust scale developed by Sampson et al. [2]. Respondents were asked how strongly they agreed (selecting from 'Don't know', 'Disagree strongly', 'Disagree', 'Agree', 'Agree strongly') that "People in this community are willing to help their neighbours", "This is a close-knit community", "People in this community can be trusted.", "People in this community generally don't get along with each other", and "People in this community do not share the same values". Items were scored from 0 to 4; low scores indicated poor social cohesion with 'Don't know'

scoring zero (as it indicated no knowledge of the community in which a person lived), through to ‘Agree strongly’ which was coded the highest at four. The last two statements were reverse coded. The second question was adapted from the reciprocated exchange scale developed by Sampson et al. [3]. Respondents answered ‘Don’t know’, ‘Never’, ‘Rarely’, ‘Sometimes’, ‘Often’ (scored 0-4 respectively) to six items, specifically “About how often do you and people in your community do favours for each other?”, “When a neighbour is not at home how often do you and other neighbours watch over their property?”, “About how often do you and people in your community ask each other advice about things such as child rearing or job openings?”, “About how often do you and people in your community visit in each other's homes or on the street?”, “About how often do you and people in your community have parties or other get-togethers?”, “About how often do you and people in your community spend leisure time together going out for dinner, to the movies, to a sporting event etc?”. The third question provided a general measure of social capital using components from Bullen and Onyx [4], with respondents answering ‘Don’t know’, ‘Not at all’, ‘Not often’, ‘Sometimes’, or ‘Yes, definitely’ (scaled 0-4 respectively) to six questions. These were “Do you feel safe walking alone down your street after dark?”, “Do you feel valued by society?”, “Do you feel there are opportunities to have a real say on issues that are important to you?”, “Can you get help from friends, family and neighbours when needed?”, “Do you help out a local group as a volunteer?”, “Do you think multiculturalism makes life in your area better?”. For all three questions an average score was generated, and higher scores indicated greater natural capital. Finally, to provide an overall estimate of social cohesion, the scores from the three scales were averaged for inclusion in analysis.

Development of vegetation data

Vegetation ratio data layers were derived from the airborne LiDAR data. The nominal vertical accuracy of the airborne LiDAR data was ±0.15m at 1 sigma and the measured vertical accuracy was ±0.05m at 1 sigma (determined from check points located on open clear ground). We used an adaptation of the method described by Miura & Jones [5] to process LiDAR data to characterise the forest structure across the city. As opposed to calculating the number of returns for each vertical layer and then divide by the total number of returns in each of the calculating units, the modified approach divides the number of returns from each vertical layer by the total number of returns below the maximum height of that layer. This

takes into consideration that the LiDAR pulse may not penetrate dense canopies. This approach calculates the proportion of LiDAR beams that are returned from within pre-determined vegetation structural layers, providing an indication of whether vegetation was present or not at that layer. The result of this processing was a binary map indicating the presence or absence of vegetation in each pixel at the specified layers. We used five vegetation layers, selected due to the fact that each could provide different ecosystem services or alter the appearance of a nature location in different ways:

- 0.15-1m; people in areas where this vegetation is present will be able to see over vegetation of this height, but are not likely to walk through it.
- 1m-2m; vegetation of this height is likely to prevent people from walking through it, and will provide a closed visual environment.
- 2m-5m; this vegetation layer will provide shade, but will not prevent people from walking through it, but also provides complexity of habitats for animals and insects.
- 5-10m;
- 10+m: these vegetation layers are likely to contribute high levels of shade to a site.

REFERENCES

1 Australian Bureau of Statistics. *Australian census*, <http://www.abs.gov.au/census> (2011).

2 Sampson, R. J., Raudenbush, S. W. & Earls, F. Neighborhoods and violent crime: a multilevel study of collective efficacy. *Science* **277**, 918-924, (1997).

3 Sampson, R. J., Morenoff, J. D. & Earls, F. Reciprocated exchange. (Chicago Neighborhood Study, Chicago, 1999).

4 Bullen, P. & Onyx, J. Measuring Social Capital in Five Communities in NSW - A Practitioner's Guide. (Centre for Australian Community Organisations and Management, Coogee, New South Wales, 1998).

5 Miura, N. & Jones, S. D. Characterizing forest ecological structure using pulse types and heights of airborne laser scanning. *Remote Sensing Of Environment* **114**, 1069-1076, (2010).
