# Supplementary material for: Health Benefits from Nature Experiences Depend on Dose
Source: Sci Rep. 2016 Jun 23;6:28551. doi: 10.1038/srep28551 (PMC4917833; doi:10.1038/srep28551)
Supplement: Supplementary Information [file srep28551-s2.pdf]

SUPPLEMENTAL INFORMATION - SURVEY

Manuscript title: Health Benefits from Nature Experiences Depend on Dose

Authors: Shanahan, D.F., Bush, R., Gaston, K.J., Lin, B.B., Dean, J., Barber, E., Fuller, R.A.

MY LIFE, MY CITY

Discovering ways to improve our city's open spaces.

Project information sheet

Many of us spend most of our time in cities. But how do city spaces influence our life-styles and wellbeing? The answer to this question will help identify ways that could make Brisbane better.

This survey is part of a research project that takes a closer look at this question. You will be asked about your use of different kinds of spaces and also about your lifestyle and wellbeing. The survey will take about 15 to 20 minutes to complete.

We do not ask for your name so you will remain anonymous. You will be asked to indicate where you live (for example, provide your address or the nearest street corner) to help characterise the open spaces in your neighborhood. Once this characterisation is done, any address information will be destroyed and it will not be passed on to anyone else.

Remember:

- Your participation is completely voluntary. If you agree to participate please fill in the consent form on the next page.
- You can withdraw from the process at any time.
- Feel free to ask any questions about the research (contact the project coordinator).

This study adheres to the Guidelines of the Ethical Review Process of the University of Queensland. If you would like to speak to an officer of the University not involved in the study, please contact the University Ethics Office on 07 3365 3924.

Consent form

Please take the time to read the project information that is provided on the previous page. Your participation is voluntary and you can choose to withdraw at any point.

You will not be asked to give your name so any information you provide is completely anonymous.

Should you wish to clarify any aspect of your potential participation or need more information you can also speak directly to a lead researcher before agreeing or disagreeing to take part in the evaluation.

If you understand the purpose of the My Life, My City research project and the nature of your involvement then please complete the following:

- ☐ I have read the information provided about the My Life, My City research project and understand the nature of my involvement. I understand any information I provide is completely confidential. I agree to take part and understand I can withdraw at anytime.
- ☐ I am over 18 years of age.

You, your home and your work

1. **What is your age range?**

   ☐ 18-20 years
   ☐ 21-25 years
   ☐ 26-30 years
   ☐ 31-35 years
   ☐ 36-40 years
   ☐ 41-45 years
   ☐ 46-50 years
   ☐ 51-55 years
   ☐ 56-60 years
   ☐ 61-65 years
   ☐ 66-70 years
   ☐ 70+ years

2. **What is your gender?**

   ☐ Female
   ☐ Male

3. **Do you reside in the Brisbane city council area?** *(market research company survey only)*
   ☐ Yes
   ☐ No

4. **Before tax, what is the *total* of all wages/salaries, government benefits, pensions, allowances and other income you *usually* receive?** (please tick one)

   ❑ $2000 or more a week ($104,000 or more per year)
   ❑ $1,500 - $1,999 a week ($78,000 -$103,999 per year)
   ❑ $1,250 - $1,499 a week ($65,000 – $77,999 per year)
   ❑ $1,000 - $1,249 a week ($52,000 - $64,999 per year)
   ❑ $800 - $999 a week ($41,600 - $51,999 per year)
   ❑ $600 - $799 a week ($32,200 - $41,599 per year)
   ❑ $400 - $599 a week ($20,800 - $31,199 per year)
   ❑ $300 - $399 a week ($15,600 - $20,799 per year)
   ❑ $200 - $299 a week ($10,400 - $15,599 per year)
   ❑ $1 - $199 a week ($1 - $10,399 per year)
   ❑ Nil or negative income

5. **What is your address?**

Remember, your responses are confidential and anonymous. Your address will only be used to map your approximate location, and will be deleted from our system once this is done. The information will not be passed on to anyone.

Street number and name (if you prefer, provide the nearest street corner or indicate your address to the nearest ten houses, e.g. 90-100 Manly Road)	

____

Suburb [ ]

Postcode [ ]

6. **How long have you lived in your current home? (please write in number)**

   Years ________________
   Months ________________

7. **Was your previous home within about 10km of your current address?**

   - ☐ Yes
   - ☐ No

8. Do you (tick any that apply):
   - ❑ Own the home you live in
   - ❑ Rent the home you live in
   - ❑ Live with parents or family
   - ❑ Board with others

   Other ___________________________

9. **Including you, how many people live at your home who are:** (please write in number)

   Under 16 years ____________________
   Over 16 years ____________________

10. **What is the level of the highest qualification or schooling year you have completed?** (please tick one)

    - ❑ Year 12 or equivalent
    - ❑ Year 11 or equivalent
    - ❑ Year 10 or equivalent
    - ❑ Year 9 or equivalent
    - ❑ Year 8 or below
    - ❑ Certificate 1, 2, 3, 4
    - ❑ Trade certificate
    - ❑ Bachelor degree
    - ❑ Associate diploma
    - ❑ Advanced diploma
    - ❑ Post-graduate degree

    ❑ Other (please specify)____

11. **Do you speak a language other than English at home?**

    - ☐ No
    - ☐ Yes

    If yes, what other language do you speak most often?

    ____

Your experiences with nature – at home

12. On an average day, about how much time do you spend at home? Please exclude the time you are asleep.

On an average work day:
- ☐ Not applicable
- ☐ Less than an hour
- ☐ 1-2 hours
- ☐ 2-4 hours
- ☐ 4-6 hours
- ☐ 6-8 hours
- ☐ 8-10 hours
- ☐ 10-12 hours
- ☐ 12+ hours

On an average non-work day:
- ☐ Not applicable
- ☐ Less than an hour
- ☐ 1-2 hours
- ☐ 2-4 hours
- ☐ 4-6 hours
- ☐ 6-8 hours
- ☐ 8-10 hours
- ☐ 10-12 hours
- ☐ 12+ hours

13. Thinking about the view from the room where you spend most of your time at home, do you have a good view of trees, parks, bushland or waterways? (please tick any that apply)

	No good view	Trees	Parks	Bushland	Ocean, lake or river
Close-by (within about 500m)	☐	☐	☐	☐	☐
In the distance (more than about 500m)	☐	☐	☐	☐	☐

14. On an average day, about how much time do you spend in the room where you can see this view?

On an average work day:
- ☐ Not applicable
- ☐ Less than an hour
- ☐ 1-2 hours
- ☐ 2-4 hours
- ☐ 4-6 hours
- ☐ 6-8 hours
- ☐ 8-10 hours
- ☐ 10-12 hours
- ☐ 12+ hours

On an average non-work day:
- ☐ Not applicable
- ☐ Less than an hour
- ☐ 1-2 hours
- ☐ 2-4 hours
- ☐ 4-6 hours
- ☐ 6-8 hours
- ☐ 8-10 hours
- ☐ 10-12 hours
- ☐ 12+ hours

15. **About how often do you usually spend more than 10 minutes in your own yard or on your deck?** (please tick one)

- ☐ I don't have a yard or deck
- ☐ Never
- ☐ Less than once a month
- ☐ 2-3 times a month
- ☐ Once a week
- ☐ 2-3 days a week
- ☐ 3-5 days a week
- ☐ 6-7 days a week

16. **Thinking about the last week, about how much time in total did you spend in your own yard or on your deck?**

- ☐ No time
- ☐ 1-30 minutes
- ☐ 31 minutes to 1 hour
- ☐ 1-3 hours
- ☐ 3-5 hours
- ☐ 5-7 hours
- ☐ 7-9 hours
- ☐ 9+ hours

Your experiences with nature – at work

17. **In a normal week, about how many hours do you spend at work?**

- ☐ No time [GO TO QUESTION 22]
- ☐ 5 hours or less
- ☐ 6-10 hours
- ☐ 11-20 hours
- ☐ 31-40 hours
- ☐ 41-50 hours
- ☐ 51-60 hours
- ☐ 61-70 hours
- ☐ 71+ hours

18. **How many days a week do you normally work?**

- ☐ 1
- ☐ 2
- ☐ 3
- ☐ 4
- ☐ 5
- ☐ 6
- ☐ 7

19. On an average week about how much of your work time do you spend outside as part of your job?

- ☐ Most of the time
- ☐ No time
- ☐ 5 hours or less
- ☐ 6-10 hours
- ☐ 11-20 hours
- ☐ 31-40 hours
- ☐ 41-50 hours
- ☐ 51-60 hours
- ☐ 61-70 hours
- ☐ 71+ hours

20. Where you work most often, do you have a good view of trees, parks, bushland or waterways? (please tick any that apply)

	No good view	Trees	Parks	Bushland	Ocean, lake or river
Close-by (within about 500m)	☐	☐	☐	☐	☐
In the distance (more than about 500m)	☐	☐	☐	☐	☐

21. On an average working day, for about how long are you in the location where you have the view mentioned above?

- ☐ Less than an hour
- ☐ 1-2 hours
- ☐ 2-4 hours
- ☐ 4-6 hours
- ☐ 6-8 hours
- ☐ 8-10 hours
- ☐ 10-12 hours
- ☐ 12+ hours

22. If you can see a park, bushland or waterway from where you work, can you name it or provide its location?

Your experiences with nature – outdoor greenspaces

23. In the last week did you spend more or less time outdoors than you usually do?

- ☐ About the same
- ☐ Less time
- ☐ More time

24. About how often do you usually visit or pass through outdoor greenspaces for any reason?
This includes, for example, beaches, bushland, playgrounds or picnic areas, dog off-leash areas, golf courses, beaches, the Brisbane River Walkway, national parks.

- ☐ Never
- ☐ Once a year
- ☐ Once every three months
- ☐ Once a month
- ☐ 2-3 times a month
- ☐ Once a week
- ☐ 2-3 days a week
- ☐ 3-5 days a week
- ☐ 6-7 days a week

25. Did you happen to visit or pass through any outdoor greenspace in the last week?
Please also think about places you may have passed through when traveling to or from places or exercising.

- ☐ No [GO TO QUESTION 26]
- ☐ Yes

26. **Over the last week, what outdoor greenspaces did you visit or pass through? Can you estimate the total time you spent there?** (please list up to seven of the places you visited for the longest time period).
This includes, for example, beaches, children's playgrounds, parks, bushland, bike-ways, picnic areas, beaches, golf courses, tennis courts and bowling greens.

Place name, location or landmark	1-29 minutes	30 minutes to - 1 hour	1-2 hours	2-3 hours	3-4 hours	4+ hours
	❑	❑	❑	❑	❑	❑
	❑	❑	❑	❑	❑	❑
	❑	❑	❑	❑	❑	❑
	❑	❑	❑	❑	❑	❑
	❑	❑	❑	❑	❑	❑
	❑	❑	❑	❑	❑	❑
	❑	❑	❑	❑	❑	❑

[GO TO QUESTION 27]

27. **Can you recall which parks, bushland or other outdoor greenspaces that you visited most often in the last year? Please estimate about how many times you visited.**

This includes, for example, beaches, picnic areas, children's playgrounds, national parks, golf courses, tennis courts, bike-ways and bowling greens. Please list up to seven locations. If you didn't visit any just leave this blank.

**Place name, location or landmark**	**1-3 visits**	**4-6 visits**	**7-9 visits**	**10 or more visits**	**Multi-day visit**	**2+ multi-day visits**
	❑	❑	❑	❑	❑	❑
	❑	❑	❑	❑	❑	❑
	❑	❑	❑	❑	❑	❑
	❑	❑	❑	❑	❑	❑
	❑	❑	❑	❑	❑	❑
	❑	❑	❑	❑	❑	❑
	❑	❑	❑	❑	❑	❑

28. Do any of the following factors prevent you from spending time in an outdoor location?
(please tick any that apply)

Factor	Never	Sometimes	Often	Most of the time
I prefer to do other indoor activities	❑	❑	❑	❑
A lack of time	❑	❑	❑	❑
Magpies or other birds	❑	❑	❑	❑
Snakes	❑	❑	❑	❑
Biting insects	❑	❑	❑	❑
Too hot	❑	❑	❑	❑
Too cold	❑	❑	❑	❑
Worried about getting too much sun	❑	❑	❑	❑
Not safe during the day	❑	❑	❑	❑
Not safe at night	❑	❑	❑	❑
No appropriate facilities	❑	❑	❑	❑
Facilities are too far away or inaccessible	❑	❑	❑	❑
A lack of transport	❑	❑	❑	❑
Poor health	❑	❑	❑	❑
Other (please specify) ________________________________	❑	❑	❑	❑

Physical activity

These questions are about different activities you might do and how often.

29. In the last week, how many times did you do any moderate or vigorous physical activity that made you breathe harder or puff or pant? (please tick all the boxes that apply)

Activity	Not at all	1 time	2-3 times	4-5 times	5-6 times	7 or more times
Individual pursuit, *e.g. jogging, cycling, swimming, tennis, surfing etc.*	❑	❑	❑	❑	❑	❑
Walking for more than 30 minutes at a time	❑	❑	❑	❑	❑	❑
Team activities, *e.g. playing or training for rugby, cricket, etc.*	❑	❑	❑	❑	❑	❑
Other activities, *e.g. gardening, heavy work around the home or at work etc.*	❑	❑	❑	❑	❑	❑

30. Excluding any walking or cycling you did for fitness or sport, in the last week how many times have you walked continuously for at least 10 minutes? (e.g. walking to the shops or around your local neighborhood)

_______ times

31. In the last week, on how many days did you exercise:

For more than 10 minutes:

☐ 0 days
☐ 1 day
☐ 2 days
☐ 3 days
☐ 4 days
☐ 5 days
☐ 6 days
☐ 7 days

For more than 30 minutes:

☐ 0 days
☐ 1 day
☐ 2 days
☐ 3 days
☐ 4 days
☐ 5 days
☐ 6 days
☐ 7 days

Your relationship with the environment

32. **Please rate the extent to which you agree with each statement. Please tick the box that indicates how you really feel, rather than how you think "most people" feel.**

	Disagree strongly	Disagree a little	Neither Agree nor Disagree	Agree a little	Agree strongly
I enjoy being outdoors, even in unpleasant weather.	☐	☐	☐	☐	☐
Some species are just meant to die out or become extinct.	☐	☐	☐	☐	☐
Humans have the right to use natural resources any way we want.	☐	☐	☐	☐	☐
My ideal vacation spot would be a remote wilderness area.	☐	☐	☐	☐	☐
I always think about how my actions affect the environment.	☐	☐	☐	☐	☐
I enjoy digging in the earth and getting dirt on my hands.	☐	☐	☐	☐	☐
My connection to nature and the environment is a part of my spirituality.	☐	☐	☐	☐	☐
I am very aware of environmental issues.	☐	☐	☐	☐	☐
I take notice of wildlife wherever I am.	☐	☐	☐	☐	☐
I don't often go out in nature.	☐	☐	☐	☐	☐
Nothing I do will change problems in other places on the planet.	☐	☐	☐	☐	☐
I am not separate from nature, but a part of nature.	☐	☐	☐	☐	☐
The thought of being deep in the bush, away from civilisation, is frightening.	☐	☐	☐	☐	☐
My feelings about nature do not affect how I live my life.	☐	☐	☐	☐	☐
Animals, birds and plants should have fewer rights than humans.	☐	☐	☐	☐	☐
Even in the middle of the city, I notice nature around me.	☐	☐	☐	☐	☐
My relationship to nature is an important part of who I am.	☐	☐	☐	☐	☐
Conservation is unnecessary because nature is strong enough to recover from any human impact.	☐	☐	☐	☐	☐
The state of non-human species is an indicator of the future for humans.	☐	☐	☐	☐	☐
I think a lot about the suffering of animals.	☐	☐	☐	☐	☐
I feel very connected to all living things and the earth.	☐	☐	☐	☐	☐

Health and wellbeing

The next few questions are about aspects of your health and wellbeing.

33. Please rate your own health.

- ☐ Very good
- ☐ Good
- ☐ Average
- ☐ Poor
- ☐ Very poor

34. What is your height?

______________cm OR ___________ feet ____________inches

35. About how much do you weigh?

____________kilograms OR ___________stone ___________pounds

36. Approximately how many times have you seen your general practitioner in the last 12 months? (please write in number)

______________ times

37. Have you ever been told by a doctor or a nurse that you have:

Condition	Yes/No		Currently under treatment or on medication?
High blood Pressure	❑ No	❑ Yes	❑ Yes
Diabetes	❑ No	❑ Yes	❑ Yes
Kidney or renal disease	❑ No	❑ Yes	❑ Yes
Heart Disease	❑ No	❑ Yes	❑ Yes
Stroke	❑ No	❑ Yes	❑ Yes
Arthritis	❑ No	❑ Yes	❑ Yes
Asthma	❑ No	❑ Yes	❑ Yes
Cancer (excluding skin cancer)	❑ No	❑ Yes	❑ Yes
Skin cancer	❑ No	❑ Yes	❑ Yes
Dementia	❑ No	❑ Yes	❑ Yes
Alzheimer’s	❑ No	❑ Yes	❑ Yes
Psychosis or schizophrenia	❑ No	❑ Yes	❑ Yes
Depression	❑ No	❑ Yes	❑ Yes
Other serious disability (please specify)______________________	❑ No	❑ Yes	❑ Yes

38. **Please read each statement and check the box which indicates how much the statement applied to you *over the past week*. There are no right or wrong answers. Do not spend too much time on any statement.**

	**Did not apply to me at all**	**Applied to me to some degree, or some of the time**	**Applied to me to a considerable degree, or a good part of time**	**Applied to me very much, or most of the time**
I found it hard to wind down	☐	☐	☐	☐
I was aware of dryness of my mouth	☐	☐	☐	☐
I couldn't seem to experience any positive feeling at all	☐	☐	☐	☐
I experienced breathing difficulty (e.g. excessively rapid breathing, breathlessness in the absence of physical exertion)	☐	☐	☐	☐
I found it difficult to work up the initiative to do things	☐	☐	☐	☐
I tended to over-react to situations	☐	☐	☐	☐
I experienced trembling (e.g. in the hands)	☐	☐	☐	☐
I felt that I was using a lot of nervous energy	☐	☐	☐	☐
I was worried about situations in which I might panic and make a fool of myself	☐	☐	☐	☐
I felt that I had nothing to look forward to	☐	☐	☐	☐
I found myself getting agitated	☐	☐	☐	☐
I found it difficult to relax	☐	☐	☐	☐
I felt down-hearted and blue	☐	☐	☐	☐
I was intolerant of anything that kept me from getting on with what I was doing	☐	☐	☐	☐
I felt I was close to panic	☐	☐	☐	☐
I was unable to become enthusiastic about anything	☐	☐	☐	☐
I felt I wasn't worth much as a person	☐	☐	☐	☐
I felt that I was rather touchy	☐	☐	☐	☐
I was aware of the action of my heart in the absence of physical exertion (e.g. sense of heart rate increase, heart missing a beat)	☐	☐	☐	☐
I felt scared without any good reason	☐	☐	☐	☐
I felt that life was meaningless	☐	☐	☐	☐

39. Do you have any allergies?

❑ No ❑ Yes, I am allergic to: ____________________________

40. How many serves of fruit do you usually eat each day?
(1 serving = 1 large fruit like apple or banana or 8 strawberries or 20 cherries or 2 smaller fruits such as kiwi fruit, apricot, plum, or 1/2 cup of juice or 4 dried apricots or 1.5 tablespoons of sultanas or 1 cup canned fruit)

☐ None
☐ Less than 1
☐ 1
☐ 2
☐ 3
☐ 4
☐ 5
☐ 6 or more

41. How many serves of vegetables do you usually eat each day?
(1 serving = 1/2 cup of cooked vegetables or 1 cup of salad or 1 medium potato)

☐ None
☐ Less than 1
☐ 1
☐ 2
☐ 3
☐ 4
☐ 5
☐ 6
☐ 7 or more

Your community

These questions are about your neighbourhood and what it is like to live there.

42. Please mark the boxes that most closely reflect how you feel.

	Disagree strongly	Disagree	Agree	Agree strongly	Don't know
People in this community are willing to help their neighbours.	❑	❑	❑	❑	❑
This is a close-knit community.	❑	❑	❑	❑	❑
People in this community can be trusted.	❑	❑	❑	❑	❑
People in this community generally don't get along with each other.	❑	❑	❑	❑	❑
People in this community do not share the same values.	❑	❑	❑	❑	❑

43. Please mark the boxes that most closely reflect how you feel.

	Never	Rarely	Sometimes	Often	Don’t know
About how often do you and people in your community do favours for each other?	❑	❑	❑	❑	❑
When a neighbour is not at home how often do you and other neighbours watch over their property?	❑	❑	❑	❑	❑
About how often do you and people in your community ask each other advice about things such as child rearing or job openings?	❑	❑	❑	❑	❑
About how often do you and people in your community visit in each other's homes or on the street?	❑	❑	❑	❑	❑
About how often do you and people in your community have parties or other get-togethers?	❑	❑	❑	❑	❑
About how often do you and people in your community spend leisure time together going out for dinner, to the movies, to a sporting event etc?	❑	❑	❑	❑	❑

44. Please mark the boxes that most closely reflect how you feel.

	Yes, definitely	Sometimes	Not often	Not at all	Don't know
Do you feel safe walking alone down your street after dark?	❑	❑	❑	❑	❑
Do you feel valued by society?	❑	❑	❑	❑	❑
Do you feel there are opportunities to have a real say on issues that are important to you?	❑	❑	❑	❑	❑
Can you get help from friends, family and neighbours when needed?	❑	❑	❑	❑	❑
Do you help out a local group as a volunteer?	❑	❑	❑	❑	❑
Do you think multiculturalism makes life in your area better?	❑	❑	❑	❑	❑

<END>
